# Glutamatergic neuron degeneration in *C. elegans* models of Frontotemporal Dementia and Amyotrophic Lateral Sclerosis

**DOI:** 10.17912/micropub.biology.002301

**Published:** 2026-08-18

**Authors:** Lexi-Amber Hassell, Mika Gallati, Monica Thoma, Selamawit Asfaw, Anne Church Hart

**Affiliations:** 1 Neuroscience, Brown University, Providence, RI, United States; 2 Carney Institute for Brain Science, Providence, RI, United States

## Abstract

Frontotemporal Dementia (FTD) and Amyotrophic Lateral Sclerosis (ALS) overlap considerably in genetic origin and pathology. Multiple *C. elegans* models of ALS/FTD have been developed, but the integrity of glutamatergic neurons in these models has not been thoroughly evaluated. Here, we report degeneration of glutamatergic phasmid neurons in animals expressing either wild-type or disease variant V337M human tau, and mild degeneration in animals expressing disease variant M337V human TDP-43. Defects caused by ectopic expression of tau were suppressed by loss of the known modifier, *spop-1* , suggesting that SPOP-1-dependent pathways are also involved in glutamatergic neuron degeneration.

**Figure 1. C. elegans models of ALS/FTD have varying degrees of dye-filling defects:** A) Four phasmid neurons in the tail dye-fill when intact. Degenerated neurons fail to dye-fill. B) This study uses animals with transgenes expressing hTau WT, hTau V337M, hTDP-43 A315T, and hTDP-43 M337V under pan-neuronal promoters. C) All animals expressing hTau have dye-filling defects suppressed by *spop-1* loss of function ( *Δ* = deletion allele, *spop-1 (dr172)* ; R414* = early stop codon, *spop-1 (bk4000)* ). Statistical values were calculated by one-way ANOVA multiple comparison tests. Statistical comparisons between N2 and Tau(low), Tau(high), or Tau(V337M) were made using only animals scored in the same trials. These were combined to make one summary panel shown here. Not shown: *spop-1* loss rescues Tau(low) and Tau(V337M) back to WT, but not Tau(high) (p<0.01). D) Animals expressing TDP-43(A315T) do not have dye-filling defects, whereas animals expressing TDP-43(M337V) have minor, but significant dye-filling defects compared to N2 animals trialed on the same days. Statistical values were calculated by unpaired t-tests. ns = p>0.05, * = p<0.05, ** = p<0.01, *** = p<0.001, **** =p<0.0001

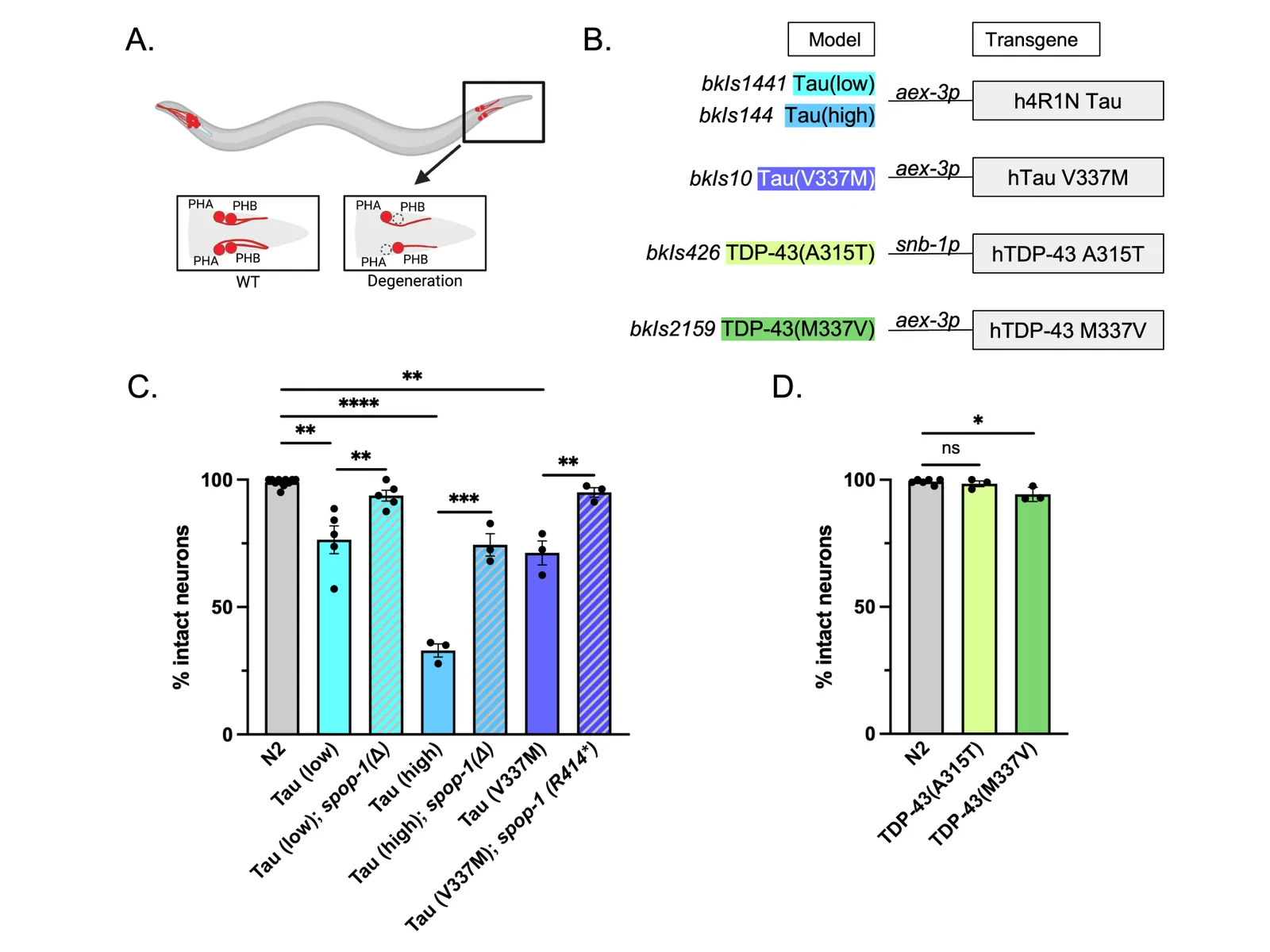



## Description

Frontotemporal dementia (FTD) is characterized by progressive cognitive decline due to atrophy of the frontal and temporal cortical neurons. Here, we focus on FTD-Tau and FTD-TDP, characterized by the accumulation of tau and TDP-43 proteins, respectively. Importantly, aberrant processing of these proteins is also associated with neurodegeneration in Amyotrophic Lateral Sclerosis (ALS) (Arai et al., 2006, Neumann et al., 2006, Agnello et al., 2021, Latimer et al., 2022, Abu-Rumeileh et al., 2025). Approximately 15% of FTD patients present with ALS, and 15% of ALS patients develop FTD, with up to 50% having cognitive symptoms (Lomen-Hoerth et al., 2002, Lomen-Hoerth et al., 2003, Burrell et al., 2016, Saxon et al., 2017). The pathological overlap of ALS and FTD suggests common mechanisms that may inform treatments to both diseases.

In ALS/FTD patients, glutamatergic cortical neurons are selectively vulnerable (Ferrer, 1999, Seeley, 2008). Previously described *C. elegans* models of ALS/FTD display locomotor defects, characteristic protein aggregation, and GABAergic neuron loss (Kraemer et al., 2003, Liachko et al. 2010, Taylor et al., 2018, Waldherr et al., 2019, Jadhav et al., 2026); however, the integrity of glutamatergic neurons has not yet been thoroughly explored in these models. Here, we assess glutamatergic neuron degeneration using a dye-filling assay.

Dye-filling is classically used to evaluate the structural integrity of glutamatergic sensory neurons in *C. elegans* . Eight bilateral pairs of chemosensory neurons–twelve amphid neurons (ADF, ASH, ASI, ASJ, ASK, and ADL) in the head and four phasmid neurons (PHA and PHB) in the tail–have exposed cilia endings that can take up lipophilic fluorescent dyes from the environment (Hedgecock et al., 1985) ( Fig. 1A ). Successful dye uptake indicates intact sensory cilia, neuronal structure and survival; failure to dye-fill usually indicates degeneration of the neuronal processes and/or cilia, or cell death ( Fig. 1A ). Dye-filling is an efficient readout of glutamatergic neuron integrity and a simple assay for investigation of underlying mechanisms. In this study, we evaluate dye-filling defects in the glutamatergic neurons of adult animals from *C. elegans* ALS/FTD models, as well as examine the impact of *spop-1* , a previously described modifier gene, on this degeneration.

**Tau model animals have dye-filling defects suppressed by *spop-1* loss of function**

Alternative splicing, hyperphosphorylation and accumulation of tau is a prominent feature of FTD, occurring in up to 50% of patients, and pathological tau is a biomarker for ALS (Stevens et al., 2019, Agnello et al., 2021, Abu-Rumeileh et al., 2025). To understand if glutamatergic neurons are vulnerable to tau toxicity, we examined three previously characterized *C. elegans* models of tauopathy. Two of the models express the wild-type 4R1N isoform of human tau pan-neuronally at different expression levels (one high and one low, Fig. 1B ). Animals from both strains exhibit tau accumulation, locomotor defects, and GABAergic neuron loss (Benbow et al., 2020, Eck et al., 2022). No degeneration of glutamatergic amphid neurons was observed in these models (Benbow et al., 2020). We examined the phasmid tail neurons in both models using dye-filling, and observed neurodegeneration, which was more severe at higher tau expression levels ( Fig. 1C ). We also examined a *C. elegans* model expressing the FTD patient variant tau V337M ( Fig. 1B, Kraemer et al., 2003), in which we also observed dye-filling defects ( Fig. 1C ). We have not examined larval animals, so the defects we observe may be developmental and not caused by age-related degenerative processes. Combined, these results demonstrate that glutamatergic neurons are defective in *C. elegans* tauopathy models, and that phasmid glutamatergic neurons are more susceptible to human tau toxicity than amphid neurons.

Genetic modifiers can reveal molecular mechanisms critical for neurodegeneration. If neurodegeneration of two different models is suppressed by loss of the same modifier gene, then the models likely share common molecular pathways involved in neurodegeneration. Loss of the conserved nuclear E3 ubiquitin ligase adaptor protein encoded by *spop-1* suppresses developmental arrest and age-dependent paralysis in a *C. elegans* C9orf72 dipeptide toxicity model of ALS (Snoznik et al., 2021). Loss of *spop-1* also suppresses GABAergic neurodegeneration, decreases accumulation of phosphorylated tau and extends life span in the Tau(high) model (Eck et al., 2022). We found that loss of *spop-1* suppresses glutamatergic neurodegeneration in all three tau models ( Fig. 1C ). Normally, SPOP-1 binds to the ubiquitin ligase CUL-3 and delivers substrates to the ubiquitin-protease pathway (Kwon et al., 2006). It remains unknown how *spop-1* loss suppresses tau-mediated degeneration, but our results, combined with previous studies, suggest a shared molecular mechanism is at work in *C. elegans* sensory and motor neurons.

**TDP-43** **model animals do not have strong dye-filling defects**

*TARDBP* variants cause ~3% of ALS cases, and TDP-43 accumulation is a common hallmark of both ALS and FTD (Majumder et al., 2018, Balendra et al., 2025). Two commonly studied patient variants–A315T and M337V–cause the protein to mislocalize from the nucleus to the cytoplasm, where it aggregates (Stallings et al., 2010). In *C. elegans* TDP-43(A315T) and TDP-43(M337V) models ( Fig. 1B ), insoluble TDP-43 accumulates in ventral nerve cord neurons (Liachko et al., 2010). Animals also show GABAergic neuron degeneration and exhibit severe, progressive locomotor impairment (Liachko et al., 2010, Jadhav et al., 2026). We observed no dye-filling defects in the phasmid neurons of TDP-43(A315T) animals and only minor dye-filling defects TDP-43(M337V) animals ( Fig. 1D ). This difference might arise from different expression levels from the transgene promoters or from a difference in toxicity of the expressed protein. Because these dye-filling defects were small (<10% loss) and inconsistent between TDP-43(A315T) and TDP-43(M337V) models, we did not examine the impact of *spop-1* loss.

**Summary**

We have established that expression of wild-type human 4R1N and mutant human V337M tau each cause dye-filling defects in *C. elegans* glutamatergic neurons. The degeneration observed in Tau(low) and Tau(high) models correlates with tau expression levels. Additionally, loss of the genetic modifier *spop-1* suppresses tau-induced glutamatergic neuron defects. This suggests that pathological tau may cause glutamatergic neuron defects via the same mechanisms by which it causes other defects. Further investigation is needed to determine why *spop-1* is required in tau-driven degeneration. Finally, defective glutamatergic neurons are not seen in all *C. elegans* ALS/FTD models. Glutamatergic neurons were intact in one of the two TDP-43 models examined. The results presented here define glutamatergic neuron defects that can be used to understand the cellular mechanisms underlying neurodegeneration in *C. elegans* models of FTD and ALS.

## Methods

Strains were maintained at 20˚C on NGM plates and OP50 *E. coli* .

**Dye-filling**

L4 animals were picked 18 hours before dye-filling to seeded NGM plates. After 18 hours, Day 1 adults were suspended on a shaker in 500 uL of DiI or DiO (final concentration 0.012 mg/mL) in M9/0.6% ethanol for 2 hours. Animals were then centrifuged at 10,000 rpm for 30 seconds and transferred to a fresh NGM plate with OP50 to recover before scoring. Animals were immobilized with 2,3-Butanedione monoxime (BDM), mounted on 2% agar pads with a glass coverslip, and visualized at 63x with a Zeiss AxioPlan 2 microscope with X-Cite 120LED Boost High-Power LED illumination. A total of 60 to 77 animals were scored per condition across 3-5 independent trials by observers blinded as to genotype.

**Statistical Analysis**

Prism 10 software was used for statistical analysis and graphics. Statistical values in panel C were calculated with one-way ANOVA and Tukey's multiple-comparisons tests. Statistical values in panel D were calculated with unpaired t-tests. Comparisons were made only between animals assayed on the same days. Results were combined into summary panels.

## Reagents

*Strain list*

**Table:** 

**Strain Name**	**Referenced in paper as**	**Genotype**	**Source**
N2	N2	*+*	CGC
CK144	Tau (high)	*bkIs144 [aex-3p::* Tau(WT 4R1N) *; myo-2p::* GFP *] V*	Taylor et al., 2018
CK1441	Tau (low)	*bkIs144 1 [aex-3p::* Tau(WT 4R1N) *; myo-2p::* dsRED *] IV*	Walherr et al., 2019
HA4811	Tau (High); *spop-1* (Δ)	*spop-1 (dr172) III; bkIs144 [aex-3p::* Tau(WT 4R1N) *; myo-2p::* GFP *] V*	This study
CK2451	Tau (low); *spop-1* (Δ)	*spop-1 ( bk3107 ) III; bkIs144 1 [aex-3p::* Tau(WT 4R1N) *; myo-2p::* GFP *] IV*	Kow et al., 2023
CK10	Tau(V337M)	*bkIs10* [ *aex-3p* ::Tau(V337M); *myo-2p* ::GFP] III	Kraemer et al., 2003
CK4000	Tau(M337V); spop-1 (R414*)	*bkIs10* [ *aex-3p* ::Tau(V337M); *myo-2p* ::GFP] *spop-1* ( *bk4000* R414*) III	This study, Kraemer Lab
CK426	TDP-43(A315T)	*bkIs426 [snb-1p::* TDP43(A315T) *; myo-2p::* dsRED *]*	Liachko et al., 2010
CK2259	TDP-43(M337V)	*bkIs2159* [ *aex-3p* ::TDP-43(M337V); *myo-3p* ::mCherry]	Jadhav et al., 2026

*Primer List*

**Table:** 

**Primer Name**	**Sequence**
spop-1 _F_external	AACCTGCCTCACAACTCATT
spop-1 _R_external	TGTAACCCTTCTGCTCATCATC
spop-1 _F_internal	CTGTGGTAGCCGAAACAGTAA

*Reagent List*

**Table:** 

**Reagent**	**Source**
1,1'-Dioctadecyl-3,3,3',3'-Tetramethylindocarbocyanine Perchlorate (DiI)	D-282 - Molecular Probes
3,3'-Dioctadecyloxacarbocyanine Perchlorate (DiO)	D275 - Invitrogen
2,3-Butanedione monoxime (BDM)	B0753 - Sigma Aldrich
1x M9 + 1mM MgSO _4_	WormBook recipe
Taq DNA Polymerase with Standard Taq Buffer	M0480X - New England Biolabs
dNTP Mix	R0192 - ThermoFisher
